# Effect of Bone Cement Implantation on Haemodynamics in Elderly Patients and Preventive Measure in Cemented Hemiarthroplasty

**DOI:** 10.1155/2015/568019

**Published:** 2015-08-30

**Authors:** Xiangbei Qi, Yingze Zhang, Jinshe Pan, Lijie Ma, Lin Wang, Jianzhao Wang

**Affiliations:** Department of Emergency Orthopaedics, Third Hospital of Hebei Medical University, Shijiazhuang, Hebei 050051, China

## Abstract

This study was to investigate the influence of bone cement implantation on haemodynamics and the preventive effect of epinephrine hydrochloride on pulmonary embolism in elderly patients with cemented semihip replacement. 128 patients were retrospectively analyzed. The patients were treated with (group A, 64 cases) or without (group B, 64 cases) epinephrine hydrochloride saline. The monitoring indicators included systolic blood pressure (SBP), diastolic blood pressure (DBP), mean arterial pressure (MAP), heart rate (HR), and pulse oxygen saturation (SPO_2_). The indicators of the two groups were compared before and 1, 2, 3, 4, 5, 6, 7, 8, 9, and 10 minutes after bone cement implantation. Analysis of variance and SNK-q test were used for the statistical analysis. Blood pressure and SPO_2_ of group B decreased with statistical difference (*P* < 0.05) and HR increased without statistical significance, comparing with those of group A. In group A, SBP, DBP, MAP, HR, and SPO_2_ after bone cement implantation did not change significantly at each time point comparing with before implantation (*P* > 0.05). Bone cement implantation has significant influence on hemodynamics in elderly patients with hemiarthroplasty. Flushing the bone marrow cavity with saline epinephrine hydrochloride is an effective measure to reduce the incidence of bone cement pulmonary embolism.

## 1. Introduction

Femoral neck fractures comprise 50% of geriatric hip fractures and are associated with radial and humeral fractures [1]. It may result from falls, especially in elderly and osteoporotic females. With the increasing of aging population, the rate of hip fractures is expected to increase from less than 2 million in 1990 to more than 6 million by 2050 [2]. Surgical management of displaced subcapital fractures of the femoral neck continues to be challenging. Internal fixation, hemiarthroplasty, and total hip replacement could be considered as appropriate solutions [3]. Total hip arthroplasty and hemiarthroplasty are effective methods to treat femoral neck fractures in elderly patients currently [4].

It is reported that, in cemented hemiarthroplasty, the right ventricular hemodynamics changed when the prosthesis is inserted into the femur. It could be detected by right ventricular ejection fraction and transesophageal dynamic electrocardiography monitoring. However, most of the patients had no clinical symptoms of hemodynamic changes, with only a small number of patients showing bone cement implantation syndrome (BCIS) [5]. BCIS is characterised by hypoxia, hypotension, and loss of consciousness occurring early after bone cementation. The postoperative perfusion lung scan and autopsy confirmed the presence of pulmonary embolism.

It has not been extensively studied how the haemodynamics change during bone cement implantation in elderly patients with femoral neck fracture and semiarthroplasty. The purpose of this study was to investigate the influence of bone cement implantation on haemodynamics and the preventive effect of epinephrine hydrochloride on pulmonary embolism in elderly patients with cemented semihip replacement.

## 2. Patients and Methods

### 2.1. Patients

The study was approved by the Ethics Committee of Third Hospital of Hebei Medical University. All signed informed consent forms were obtained. 128 patients (54 males, 74 females; average age of 83.5, range of 75–92) with femoral neck fracture who were admitted to the hospital from January 2008 to January 2012 were enrolled in the study. The inclusion criteria were patients with Garden 3 or Garden 4 of acute hip fractures and patient age >70. The patients with hypertension, diabetes, or lung disease were excluded.

All patients underwent bone cement-hemiarthroplasty using Palacos bone cement (Heraeus Medical, Berlin, GER). The patients were treated with epinephrine hydrochloride saline (Hefeng Pharmaceutical Co., Ltd., Shanghai, China) (group A, 64 cases) or not treated (group B, 64 cases).

### 2.2. Surgical Process

Hemiarthroplasty was performed as described previously [6]. Briefly, the patients were anesthetized with tracheal intubation methods, and the lateral Hardinge incision was used in all the patients' cases. The medullary cavity was flushed with normal saline (group B) or epinephrine hydrochloride (1 : 500000, group A) followed by dry gauze packing. The bone cement was implanted into the bone marrow cavity when it became doughy. Their vital signs were closely monitored during the surgery.

### 2.3. Monitored Indicators

The electrocardiogram monitor (M8004A, Philips Healthcare, Netherlands) was used to detect the blood pressure, heart rate (HR), electrocardiogram (ECG), and pulse oxygen saturation (SPO_2_) in all patients. The monitored indicators included the systolic blood pressure (SBP), diastolic blood pressure (DBP), mean arterial pressure (MAP), HR, and SPO_2_. The values before using bone cement and 1, 2, 3, 4, 5, 6, 7, 8, 9, and 10 min after bone cement implantation were recorded.

### 2.4. Statistical Analysis

All data was analyzed by SPSS 13.0 software. The indicators at different time points were analyzed by multifactor analysis of variance (ANOVA). If it was statistically significant, the SNK-q test was used for comparisons between the two groups. *P* < 0.05 was considered as statistically different.

## 3. Results

### 3.1. Indicator Changes in Group B

All patients showed decrease of blood pressure 1 min after implantation. 2–6 min after implantation, the blood pressure began to decrease significantly (SBP and DBP, *P* < 0.01) compared with that before implantation. About 7 min after bone cement implantation, the blood pressure began to rise and returned to normal 10 min after implantation.

**Figure 1:**
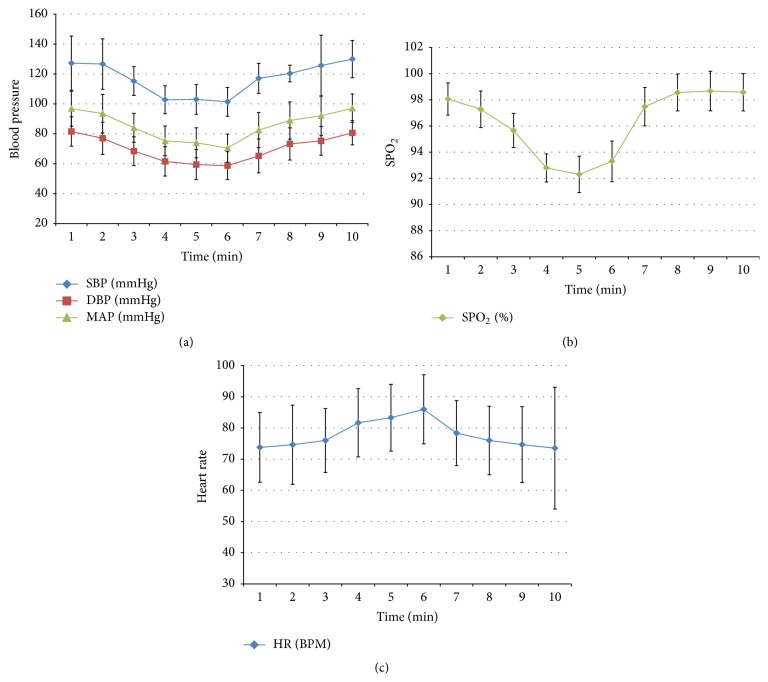
Indicator changes in patients of group B. (a) Blood pressure changes after implantation of bone cement, (b) changes of SPO_2_ after implantation, and (c) changes of heart rate after implantation.

As for blood pressure decrease (Figure 1(a)), 35 patients (35/64, 54.7%) presented with a decrease range within 10 mmHg, 24 patients (24/64, 37.5%) presented with a decrease range of 10–20 mmHg, and 5 patients (5/64, 7.8%) presented with a decrease range of more than 20 mmHg. SPO_2_ (Figure 1(b)) decreased from 99.65 ± 0.35% to 92.80 ± 1.08% with statistical difference (*P* < 0.05) 1 min after bone cement implantation. It reached the lowest point 4 min after implantation and began to rise about 5 min after implantation and returned to normal 10 min after implantation. HR increased slightly without statistical significance (*P* > 0.05) (Table 1 and Figure 1(c)).

### 3.2. Indicator Changes in Group A

**Figure 2:**
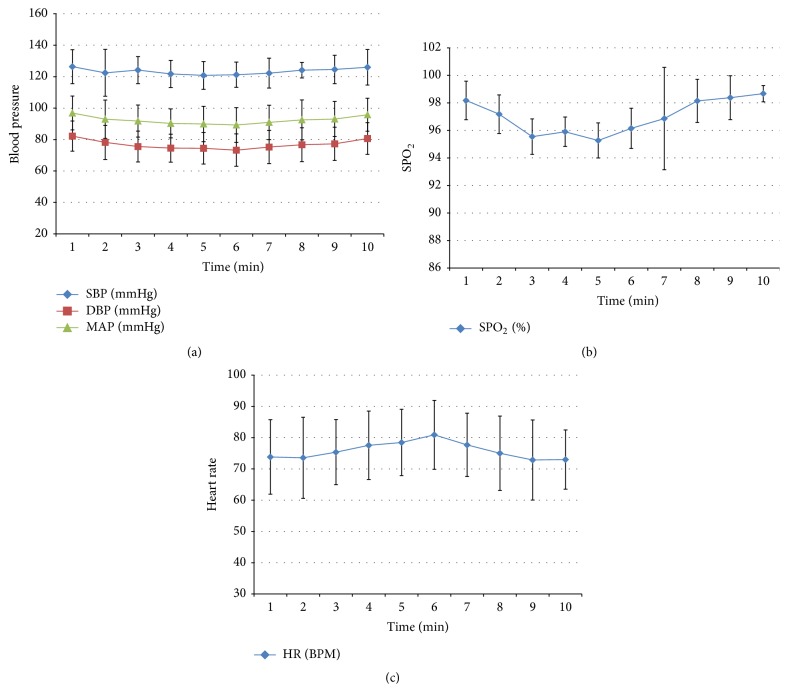
Indicator changes in patients of group A. (a) Changes of blood pressure after implantation of bone cement, (b) changes of SPO_2_ after implantation, and (c) the change of heart rate after implantation.

In all patients of group A, SBP, DBP, MAP, HR, and SPO_2_ did not change significantly at each time point before or after bone cement implantation (*P* > 0.05). The mild blood pressure decrease occurred in 3 patients (SBP decrease of 9 mmHg, 8 mmHg, and 10 mmHg, resp., and DBP decrease of 10 mmHg, 8 mmHg, and 7 mmHg, resp.). One case showed arrhythmia (ventricular tachycardia) (Table 2 and Figure 2).

## 4. Discussion

Currently, the application of bone cement prosthesis to elderly patients with femoral neck fracture is quite popular. However, there are still many clinical issues in bone cement materials and technology [7]. Pulmonary embolism is often reported in a variety of clinical studies and animal experiments of hemiarthroplasty [8–10]. The aetiology and pathophysiology of BCIS are not fully elucidated. Several mechanisms have been proposed. Initial theories focused on the release into the circulation of cement monomer during cementation. Some recent studies have investigated the role of emboli formed during cementing and prosthesis insertion. Besides, there are several other mechanisms such as histamine release, complement activation, and endogenous cannabinoid-mediated vasodilatation [11, 12].

In the present study, the patients without treatment of epinephrine hydrochloride displayed blood pressure decrease in varying degrees after bone cement implantation. SPO_2_ decreased significantly, indicating that the bone cement implantation had a significant influence on haemodynamics. It could be attributed to multiple factors. In in vitro experiments performed by Kim et al. [13, 14], the results showed that the methyl methacrylate monomer could directly inhibit the myocardium through inhibiting the uptake of sarcoplasmic reticulum Ca^2+^, with a dose dependent manner. Helgason [15] considered that the stasis of blood flow, the damage of vascular wall, and the change of blood components may cause thrombosis. After bone cement monomer penetrating into the blood, it makes blood be in the hypercoagulable state and promotes the platelet aggregation. Simultaneously, the heat from polymerization of monomers could damage the endothelial cells and lead to local thrombosis. Bone cement monomer could also act on the calcium channels of vascular smooth muscle, causing angiectasis, blood siltation, and blood pressure decrease [16]. Dahl [17] found that after the injection of bone cement, a large number of bone cement monomers enter into the blood (reach to 3599 ng/mL, after 30 s) and these bone cement monomers could activate the coagulation system and produce thrombin from the pulmonary capillary bed. Then, platelet aggregation, thrombosis, and lung embolism may occur.

In our study, we also found that, in patients without treatment of epinephrine hydrochloride, blood pressure decreasing (5–45 mmHg) was most obvious 2~6 min after bone cement implantation, SPO_2_ decreased significantly, and HR increased slightly. Maybe it was due to the toxic effects of bone cement monomer, which led to the formation of tiny blood clots and caused a mild pulmonary embolism. Thus, it resulted in peripheral blood pressure decrease and the compensatory increase of the heart rate in order to increase cardiac output. Moreover, the bone cement could directly inhibit myocardium, with the decreased myocardial contractility or the abnormalities of conduction system. It may result in decreased cardiac output or arrhythmia, which further exacerbates the decrease of blood pressure. Memtsoudis et al. [18] considered that the occurrence of fatal cardiovascular complications may be associated with the combined action of a number of factors such as hypovolemia, myocardial dysfunction, arrhythmia, embolism, and histamine release. In elderly patients, the function of the systemic organ declined, especially their cardiopulmonary functional reserve, was insufficient, with low surgical tolerance. Their body is slow to respond to the hypotension and hypoxemia caused by bone cement implantation and cannot tolerate the hemodynamic changes and temporarily affect the cardiorespiratory function.

Modern bone cement technique obtains the maximum degree of bone cement interlocking effect through the use of low-viscosity bone cement and compression techniques. However, the application of this technology could produce great intramedullary pressure in the femoral bone marrow cavity. This pressure exceeds the pressure in the general venous circulation, causing the rupture of intramedullary blood vessel with thin wall. Rupture of blood vessels could make the intramedullary fat. Moreover, it may lead to bone marrow, bone debris, and bone cement particles entering into blood vessels and bloodstream and through the metaphyseal veins. Embolism or air embolism is formed due to heat expansion of gases into the blood circulation system [19]. In the present study, we found that the decrease of blood pressure and SPO_2_ are not obvious when we expanded and rasped the medullary cavity. Implantation of bone cement and prosthesis pressurizing (4–6 min after bone cement implantation) could significantly decrease the blood pressure and SPO_2_. The production of the enormous pressure is not clearly elucidated. It is probably due to the fact that the insertion of femoral stem into the medullary cavity leads to the tiny blood clots, fat drops, bone marrow composition, bone cement particles, and other materials squeezing into the venous cavity. They can form many small emboli and subsequently cause multiple embolism of small branches of pulmonary artery and the gas exchange dysfunction, followed by pulmonary embolism. This speculation was reported in the previous study [20].

In the present study, there were 3 cases of blood pressure decrease and 1 case of arrhythmia in group A. We speculated that the 4 patients had mild bone cement pulmonary embolism. In group A, the medullary cavity was flushed with saline epinephrine hydrochloride and packed with the gauze dipped with saline epinephrine hydrochloride. Firstly, epinephrine can constrict blood vessels to maintain the blood vessel tension, increase the serum sodium level, and enhance myocardial tension to restore the effective circulating blood volume and increase the blood pressure. Secondly, epinephrine can reduce the absorption reaction of the bone cement monomer against the toxic effects of bone cement monomer through constricting blood vessels. Thirdly, epinephrine can constrict the small blood vessels in the bone marrow cavity, which reduces the chance of the air, fat, bone marrow, blood clots, and bone cement particles entering into the blood circulation system. Thus, it greatly reduces the incidence of pulmonary embolism caused by bone cement. Therefore, flushing the bone marrow cavity with saline epinephrine hydrochloride and packing the bone marrow cavity with the gauze dipped with the saline epinephrine hydrochloride could reduce the incidence of bone cement pulmonary embolism.

## 5. Conclusions

In summary, bone cement implantation has significant influence on hemodynamics in elderly patients with hemiarthroplasty. Flushing the bone marrow cavity with saline epinephrine hydrochloride is an effective measure to reduce the incidence of bone cement pulmonary embolism.

## Figures and Tables

**Table 1:** The change of hemodynamics after implantation of bone cement in group B (*N* = 64, mean ± SD).

		SBP (mmHg)	DBP (mmHg)	MAP (mmHg)	HR (BPM)	SPO_2_ (%)
Before using bone cement		130.26 ± 19.04	82.74 ± 7.75	98.58 ± 11.51	72.69 ± 10.19	99.65 ± 0.35

After using bone cement (min)	1	127.26 ± 18.06	81.55 ± 9.81	96.79 ± 12.56	73.78 ± 11.19	98.07 ± 1.22
	2	126.67 ± 16.86	76.96 ± 10.84	93.53 ± 12.85	74.64 ± 12.69	97.28 ± 1.39
	3	115.28 ± 09.67	68.45 ± 9.59	84.06 ± 9.62	75.99 ± 10.24	95.65 ± 1.30
	4	102.76 ± 09.36	61.55 ± 9.81	75.29 ± 9.93	81.66 ± 10.93	92.80 ± 1.08
	5	102.98 ± 09.95	59.45 ± 10.01	73.96 ± 10.03	83.31 ± 10.73	92.30 ± 1.39
	6	101.38 ± 09.60	58.75 ± 9.28	70.46 ± 9.39	85.99 ± 11.06	93.30 ± 1.57
	7	117.05 ± 10.03	65.25 ± 11.29	82.52 ± 11.71	78.34 ± 10.42	97.48 ± 1.46
	8	120.34 ± 05.56	73.21 ± 10.77	88.92 ± 12.41	75.99 ± 11.00	98.56 ± 1.41
	9	125.63 ± 20.31	75.28 ± 9.61	92.06 ± 13.17	74.67 ± 12.18	98.68 ± 1.51
	10	129.94 ± 12.47	80.67 ± 8.09	97.09 ± 9.55	73.51 ± 19.52	98.57 ± 1.43

*F*		43.37	54.53	51.21	8.62	25302.70

*P*		0.000	0.000	0.000	0.000	0.000

MS group		185.89	95.42	126.46	146.44	1.75

**Table 2:** The change of hemodynamics after implantation of bone cement in group A (*N* = 64, mean ± SD).

		SBP (mmHg)	DBP (mmHg)	MAP (mmHg)	HR (BPM)	SPO_2_ (%)
Before using bone cement		128.36 ± 8.94	82.36 ± 9.72	97.69 ± 9.98	73.00 ± 8.91	99.50 ± 1.21

After using bone cement (min)	1	126.36 ± 10.84	82.18 ± 9.59	96.90 ± 10.74	73.82 ± 11.91	98.18 ± 1.40
	2	122.45 ± 14.94	78.18 ± 10.84	92.94 ± 12.21	73.55 ± 12.96	97.18 ± 1.40
	3	124.18 ± 8.66	75.55 ± 9.81	91.76 ± 10.19	75.36 ± 10.42	95.55 ± 1.29
	4	121.73 ± 8.63	74.55 ± 8.91	90.28 ± 9.19	77.55 ± 10.93	95.90 ± 1.07
	5	120.82 ± 8.84	74.45 ± 10.01	89.91 ± 11.21	78.45 ± 10.61	95.27 ± 1.27
	6	121.25 ± 8.03	73.25 ± 10.29	89.25 ± 11.04	80.88 ± 11.03	96.15 ± 1.46
	7	122.25 ± 9.47	75.25 ± 10.55	90.92 ± 10.91	77.68 ± 10.13	96.86 ± 3.72
	8	124.14 ± 4.95	76.71 ± 10.77	92.52 ± 12.71	75.00 ± 11.90	98.14 ± 1.57
	9	124.57 ± 9.03	77.29 ± 10.61	93.05 ± 11.14	72.85 ± 12.81	98.38 ± 1.59
	10	126.00 ± 11.31	80.67 ± 10.07	95.78 ± 10.48	73.00 ± 9.47	98.67 ± 0.59

*F*		3.87	6.33	4.37	3.81	45.28

*P*		0.000	0.000	0.000	0.000	0.000

MS group		94.22	102.45	119.49	122.68	2.83
